# The role of internet resources in health decision-making: a qualitative study

**DOI:** 10.1177/2055207619888073

**Published:** 2019-11-07

**Authors:** Lauren Georgia Bussey, Elizabeth Sillence

**Affiliations:** 1 School of Social Sciences, Humanities and Law, Teesside University, Middlesbrough, Tees Valley, UK; 2 Psychology and Communication Technologies Lab, Department of Psychology, Northumbria University, Newcastle upon Tyne, UK

**Keywords:** Decision-making, internet, health information, personal experiences, distributed, eHealth, communication, healthcare professional

## Abstract

**Objective:**

Internet resources remain important for health information and advice but their specific role in decision-making is understudied, often assumed and remains unclear. In this article, we examine the different ways in which internet resources play a role in health decision-making within the context of distributed decision-making.

**Methods:**

We conducted semi-structured interviews with 37 people in the United Kingdom who reported using the internet in relation to decision-making, and representing a range of long- and short-term health conditions. The interviews focused on decision-making activities across different settings and in relation to different stakeholders to understand how internet resources play a role in these activities. We carried out a thematic analysis of the interviews.

**Results:**

We identified three main ways in which internet resources played a role in health decision-making. A supportive role (as a decision crutch), a stimulating role (as a decision initiator), and an interactional role (impacting on the doctor–patient relationship). These three roles spanned different resources and illustrated how the decision-making process can be impacted by the encounters people have with technology – specifically internet based health resources – in different ways and at different time points.

**Conclusions:**

Examining health decisions with respect to internet resources highlights the complex and distributed nature of decision-making alongside the complexity of online health information sourcing. We discuss the role of internet resources in relation to the increasing importance of online personal experiences and their relevance within shared decision-making.

## Introduction

Patient involvement in health decisions is widely regarded as a marker for quality healthcare.^1^ The accessible nature of the internet and the proliferation of social media and peer resources have afforded users the opportunity to find and share experiential and anecdotal knowledge surrounding health and wellbeing.^2^ This collaborative knowledge building means that patients have access to information about a variety of health decisions,^3^ and this information can be used to prepare for, or to complement, healthcare appointments.^4^

Greater patient involvement has been shown to lead to better decision-making outcomes.^5^ Patients are able to ask more questions,^6^ and are better equipped to collaborate with a healthcare professional around health information.^7^ Alongside this increased level of patient involvement, we are seeing healthcare professionals taking an increasingly flexible approach to interactions with patients. Together, these changes epitomize a shift from the traditional paternalistic healthcare model whereby patients complied with health professionals’ recommendations, to one of mutual participation and shared health decision-making.^7–9^ Here decisions are made in a collaborative way, and the concerns and contexts of individuals and their families play a key role. A central tenant of shared decision-making is having access to the information necessary to engage in discussion of options and preferences. Traditionally, shared decision-making has been understood and studied within the context of the consultation room.^10^ In such cases, patients are typically involved in decisions about treatment options and engage in one-off dyadic encounters with their healthcare professionals. Decision-making activities, however, are more complex and varied, and rather than occurring in discrete, single encounters are likely to be shaped over time through exposure to and reflection on a range of encounters with resources. Different resources (informational and supportive) present in different ways and at different time points for people making decisions. Internet resources for health information and advice can be viewed within this more distributed notion of decision-making.

With 72% of US users and 75% of UK users typically searching online for illness, treatment, and medical procedure advice,^11,12^ the internet has been hailed as a catalyst for patient power.^13^ The asynchronicity and privacy afforded by the online environment enables discussion of sensitive topics and provides geographically and socially isolated individuals with the opportunity to participate in health talk.^14^ When faced with a health concern, people are motivated to self-diagnose and source more information about the illness.^15^ This information may include traditional factual or statistical online information sources, as well as the lived experiences of others.^16,17^ Information based on personal experiences is popular, and conveys social and emotional information regarding the processes and outcomes of decisions that help identify decisions to be made and the available alternatives.^1^

The literature on the internet and health decision-making suggests that a range of web-based resources can be useful in supporting decision-making processes,^18^ and influencing final decisions.^19^ Online support groups (OSGs) and curated peer resources support increased confidence and empowerment around decision-making,^20^ offer opportunities to identify options,^16^ are relied upon to help inform treatment choices,^21,22^ and allow people to reflect on decisions already made.^23^ Hypothetical decision-making tasks also confirm the benefits of providing people with narratives to aid their understanding of treatment experiences and fuel additional information-seeking behaviors.^24^ In reality, people are likely to be exposed to both fact based and patient experiences style information,^25^ and will make use of both in different ways. Using factual information, for example, to underpin certain decisions and personal experiences to add contextual and experiential detail to weigh up different options.^1^

While this existing body of work seeks to differentiate between types of information or type of website or platform on decision making activities, our article adds to the literature on decision-making in health by taking a broader, more holistic approach to the context of decision-making. We do not target specific decisions (or activities) or individual health conditions. Instead, we aim to examine the role of internet resources within the lens of distributed decision-making. This concept captures the notion that decisions are shaped over time through knowledge and encounters, and extend beyond the consultation room. Decisions are unlikely to be discrete events but are ongoing and revisited over time. This will vary depending on the nature of the health condition and thus lead to differences in timescales, additional resources and supporting characters (health professionals, family and friends).^27^ Rapley’s ideas are in no way prescriptive but simply allow attention to be focused on the varied and integrated ways in which internet resources may play a role in decision-making around health. Taking this approach to understanding the role of internet resources is novel, and builds upon Rapley’s concept of multiple sources of knowledge. This means that we can contextualize the role of internet resources more realistically across time, across stakeholders and, where appropriate, beyond single doctor–patient encounters.

In taking this approach, we sought to capture the extent to which individuals recognized and valued internet resources in this context. We consciously use the term ‘internet resources’ in this study to capture places that people use for information, advice, interest and support. These places may be actively sought or simply encountered whilst online and in using this term our intention here was two-fold: Firstly, to recognize that online health information sourcing is complicated.^26^ Indeed, information seekers often conflate, simplify, forget or are untroubled by the specific source of the information online. This can lead to a range of different source attributions spanning a range of levels of specificity, of which a commonly accepted and understand term is ‘the internet’ even if often misrepresented from a technical standpoint. Secondly, to avoid unintentionally leading participants to talk solely about a specific site or social media platform, or to feel inhibited from discussing more than one source of information or refraining from discussing a site that was perceived to be of poor quality or if participants had simply forgotten the name(s) of the sites(s). Finally, avoiding specific sites and platforms avoids implying value judgements or restrictions around what is deemed ‘information’ versus ‘support’ or ‘advice’, and allows us to consider the role of internet resources (both factual and experiential) across health decision-making.

Opening out decision-making to a range of health conditions (both long- and short-term) allows us to identify the more fundamental and broad ways in which internet resources can be impactful upon decision-making free from any specific factors and constraints imposed by a particular condition or type of decision. In fact, we allow people to report their own experiences and meanings of decision-making and how internet resources related to that. Furthermore, we examine the whole context in which the decision-making took place and take a distributed decision-making approach to see how internet resources played a role within those spaces, over time and in relation to other key stakeholders.

## Materials and Methods

### Sample and recruitment

This study received ethical approval from (blind for review Ethics Committee). We conducted semi-structured interviews between 5 February 2016 and 17 October 2016 with people who self-reported their use of the internet in health decision-making. Participants were recruited using email distribution lists, social media, and poster advertisements across the University campus and local town centre coffee shops.

### Data collection

Participants received a full description of the study information, and provided informed written consent before taking part. Prior to interviews, participants completed a ‘health paragraph’ detailing their use of the internet in relation to the health issue. This helped confirm participant eligibility, but was primarily used to develop contextual detail for the interviews.

With the exception of one Skype call, all interviews were conducted face to face by LGB, a researcher with experience of qualitative interviews and a background in health psychology. The semi-structured interviews lasted between 20 and 60 min, were conducted at the University, and centred on an interview guide developed by the authors. The interview guide began by exploring the health condition described in the participant’s written health paragraph. Questions covered participants’ experiences of their health condition or issue, their contact, if relevant, with healthcare professionals, and their search and selection of health related information on the internet. Participants were asked to describe the decisions they had faced in relation to this health issue, and to think about the information and the other people they had used to support this decision-making. We specifically used the term ‘internet’ when talking to participants so as not to lead towards specific websites, social media platforms or forums. The guide was used flexibly, with questions being omitted, added, adapted and elaborated according to each participant’s response to ensure participants’ experiences shaped the content and direction of the interview. All interviews were audio recorded and transcribed verbatim and pseudonyms applied to the data.

### Data analysis

Both authors began by reading and re-reading the transcripts. We then identified passages in which participants discussed their use of the internet in relation to decision-making. These passages occurred in a number of different places throughout the transcripts. Firstly, we were able to identify a number of extracts in which participants provided an account of their health decision-making, describing the ways in which they engaged with online resources as part of that process. This was typically in response to a direct question about the role of the internet in health decision making. Secondly, we were mindful of the fact that some participants’ accounts of decision-making actually emerged through their discussion of their encounters with healthcare professionals or through a detailed discussion of their internet use and habits. Some of these discussions began more broadly and covered issues of interest but not directly relevant to the study’s focus, for example, changes in the nature and volume of health web resources available, or difficulty in making appointments. We therefore focused on those further instances in which talk of the internet emerged in relation to their decision-making.

For both cases, we examined the extracts taking careful note of the position of internet resources in relation to the decision-making activity. We noted, for example, whether the internet use appeared to precede or follow discussion of the decision and documented the reported internet activity, i.e. to seek or verify information or to ask for opinions or advice. We sought to situate the internet activity in relation to other resources and stakeholders and asked what decisions are being described, and what value or significance is assigned to internet resources in relation to the decision-making process. We were careful not to make assumptions about the positive or negative role of internet resources, and noted that accounts often explicitly rejected or refuted the direct role of the internet in decision-making. We also took a broad perspective on decision activities as described by participants. We began by grouping extracts around time points, decision activity and stakeholders. We then looked across these groupings for constructs that captured the way in which internet resources played a role in decision-making. We were then able to group examples together under three main headings that noted the importance of: (a) the supportive role of internet resources, (b) the stimulating role of internet resources and (c) the interactional role of internet resources. Recruitment ended when data saturation was achieved and no further roles were determined from the analysis.

## Results

A total of 29 females and 8 males (age range 18–66 years, mean 29.10 years, SD = 12.16) participated in the study with all but one residing in the North East of England. Of these volunteers, 15 (2 males) (age 18–66 years, mean 33.53 years, SD = 14.61) had self-reported long-term or chronic health conditions (LTHC; see Table 1 for details) and the remaining 22 volunteers (6 males) (age range 18–50 years, mean 25.75 years, SD = 9.14) had experience of a number of short-term health concerns (STHC), including upper respiratory tract infections, cystitis, and muscular pains.

**Table 1.** Breakdown of participants with long-term health conditions.

Health condition	Description/comments	Total number of participants (*N* = 15)
Pregnancy(stage of life)	2 participants were pregnant for the first time1 participant had one previous miscarriage, and 1 participant was expecting her second child	4 female(Participants: Sarah, Jodie, Emily, Amy)
Digestive health conditions	2 Participants had Ulcerative Colitis, 1 had Crohn’s disease, and the remaining 3 had Irritable Bowel Syndrome	4 female, 2 male(Participants: Aria, Andrew, Hannah, Erin, Zoey, Jake)
Hormone conditions	1 Participant had Hypothyroidism, 1 Participant had Polycystic Ovary Syndrome1 Participant had Type 2 diabetes	3 female (Participants: Jayne, Gabbie, Leah)
Skin condition	1 Participant had Eczema	1 female (Alanah)
Autoimmune disorder	1 Participant had Sjögren's syndrome	1 female (Debbie)

Most participants began by describing their health condition or the issue of concern and their perspectives on the decisions it generated. They talked about their use of the internet with respect to the health condition, the sites they had visited, and their interactions with healthcare professionals. They talked about their perspectives of the process of using the internet in relation to their health decision-making and finally reflected on their overall satisfaction with their decisions. Rapley asserts that decision-making is never just a solo, cognitive activity, but is instead distributed over a range of people and that the process of decision-making is ‘initiated, sustained and transformed’ over a range of encounters with both people and technologies.^10^ In this article, we pay close attention to the encounters people have with technology – specifically web based health resources – in order to understand the way they shape decision-making. We examine how those encounters inform decision-making activities and their relation to healthcare professionals.

### Decision activities

Participants engaged with a wide range of activities related to decision-making. Many of these were extremely tangible and straightforward and others less so and often difficult to describe. Participants sometimes had difficulty articulating the decision and often found it difficult to pinpoint the point or points in the process where decisions had been made. Allowing people to define and describe the way in which they used the internet in relation to their health highlighted a number of self-reported decision activities. People described straightforward decisions around treatment but also decisions about testing, changes to medication, and alternative products. Decisions around the acceptance of health status and identity were also discussed.

### Internet resources and their role in decision-making

We identified three main ways in which internet resources provided support to health decision-making. These three forms of resource provided succour to the decision-making process in different ways and at different time points, and in some participants’ accounts more than one of these resource types was present. The overarching aim here was to note the main ways in which internet resources played a role for people at specific points in their own decision activities. Examining these three roles: supportive role, stimulating role and interactional role, allowed us to see the differences and indeed the tensions around the way people describe their relationship with the internet in this context.

### A supportive role: provides a crutch for decision-making

For some participants, it was clear that internet resources were always involved to some extent in decision-making although these resources were not the starting point for the decision nor were they the primary source of knowledge. The decision may have started elsewhere, for example, in the presence of the healthcare professional. At a later point, away from that setting, and often involving other stakeholders such as family or partner, internet resources then provide support to a decision that has often been formed or even undertaken elsewhere. In these cases, the supportive role of internet resources acted as a form of crutch, an aid to decision-making rather than being instrumental to decision-making. The resources are used alongside the decision-making process and support decisions made already. In this way, internet resources provide a reality check, they offer information to support the process of decision-making and experiential information to aid understanding of the experience of making the decision and the consequences. In discussions of this kind, internet resources and the decision seem quite separate in many ways, or at least operate in parallel. In some cases, the internet is rejected explicitly from being directly involved in the decision-making (as in the quote from Jodie) ‘regardless of what it said’.

Jodie, LTHC: ‘Started looking up the test [Down’s Syndrome Test] that they do from that point on to try and find out what it involved erm, so, I think I first went to like the NHS’ pages itself and that, that was quite comprehensive then they had links to like external organisations that were talking about this test so, erm, it’s like an invasive test so they have to stab you with a big needle so we were quite apprehensive about risk and things so that, that was kinda the stuff we were researching so not what the test involved but the risk factor and trying to weigh up whether it was worth having the test, so spent a lot of time looking online and then just decided that regardless of what it said on the internet we’d probably want to know either way, so went ahead with that … for serious stuff like having the genetic testing worrying about risk factors I was looking at NHS and their affiliated websites, but I spent quite a lot of time looking at forums of people who’d already had the test … find out how hideous it was how painful it was, or so not necessarily to help me make a decision to have it or not, but the kind of less credible websites I was using as like a secondary resource to find out about the experience of others … Yeah so I wasn’t kind of taking anybody’s advice on board and thinking right they’ve had it done so I should, it was more you know there’s a bunch of women that said it isn’t that bad so it makes it more easy to make the decision …’

Here, Jodie recalls her use of two different kinds of internet resource in providing support to her decision-making. The first sees the participant refer to the NHS pages as a way of gauging the facts about the test. Here, there is a clear focus on the risk associated with the procedure and we hear that the participant and her husband were trying to weigh up the potential risks of the tests (this appears to be a process occurring alongside or parallel to the internet use itself). In fact, the internet as a resource for this part of the decision-making process is explicitly rejected and the decision occurred in spite of any information found online. Jodie also refers to her use of online forums and makes clear that reading other people’s accounts is not necessarily connected to decision-making directly but does make the decision-making process easier. In this example, the couple have made the decision already but hearing from other women about their actual experiences of the procedure supports the process, and helps the couple feel comfortable with the decision they have made.

This example highlights the distributed nature of decision-making. The role of internet resources is to provide a support to a decision that was undertaken at a different time and place in the presence of the healthcare professional. The support comes through using more than one website or platform, and again that distributed nature is captured in the language of the participant as they variously refer to their use of internet resources as ‘looking up, NHS pages, external organisations, the internet, looking online, affiliated websites, forums, less credible websites.’ Taken as a whole, the role of these internet resources was to provide support to a decision already made.

This sense that internet resources act as a crutch to support decision-making was also described by participants in relation to their health decision-making more generally. Again, even without identifying a particular decision, there was a feeling that internet resources provided support around the decision-making process as a whole rather than having a direct influence on the decision itself. This can be seen in the quotes below where internet resources are roundly described in a positive, almost soothing manner by Amy and by Jake, who initially describes the online experiences as forming his decision but then corrects himself to report that experiences help ‘support’ decisions.

Jake, LTHC: ‘So that’s why when I do look at their experiences it does form me decision it just kind of like no, helps support it if that makes sense? …’

Amy, LTHC: ‘I think online searching makes my decision-making more easier and it relaxes me because otherwise if I didn’t know what would happen I would worry because my family is not here and I only have a few friends here, if I feel alone I would worry definitely.’

### A stimulating role: initiates decision-making

In the second category, internet resources play a more active role in decision-making. Here, participants actively use internet resources to look for information in order to initiate decision-making or through their online engagement come to realise that there is a decision to be made. The role of the healthcare professional varies in relation to this process. Sometimes they appear as a central character, one that is incorporated into the decision-making process, and other times appear almost irrelevant – someone who will simply be ‘told’ the result of the decision. In all these cases, the participant themselves uses the internet resources to initiate the decision process rather than it being something that is driven by the healthcare professional. In this way, the importance of the distribution across time is apparent. Encounters with healthcare professionals may not trigger decision-making, but later engagement with internet resources may do so. Alternatively, decisions made previously with the healthcare professional can be updated and transformed over time after encounters with different stakeholders and internet resources.

So, in the first example (Emily), we observe the contrast between the various stakeholders in the decision-making process; the calm midwife (who does not seem to have initiated decision-making, at least not explicitly) versus the girls with lots of information on Facebook. This is followed by Emily explicitly linking the online information to the decision to return to the midwife and ask for the blood test. Interestingly, the midwife acts to sustain the decision about the blood test, despite it not being a decision she initiated.

Emily, LTHC: ‘So when the midwife told me about that [the potential for complications arising from the participant’s blood type] she seemed very calm and you know all that kinda stuff, but when I asked the girls on the Facebook group they were like, they told me a lot more than she did and it was from their information I went back to my midwife and say well look I’ve found out this kind of information so can we progress with having my partners blood tested, what blood type he is then we’ll progress from there… so the minute I knew I could have ***** tested and I said that to her she could almost, she was in agreement with me from the minute that I mentioned it. You know whilst I don’t think if I didn’t know about that blood test then she probably wouldn’t have suggested it to me I don’t think… Yeah, I think when you’re armed with more information you almost get more information back.’

Again, in the second example (Jayne) we see internet resources playing a direct role in initiating decision-making. In this case, the decision itself was one originally made alongside the healthcare professional but an encounter with *‘stuff I found online’* prompts Jayne to update her decision. The information the participant has found becomes the impetus to initiate a conversation with the specialist about reducing medication. The participant describes the power that comes with the realization that other people were experiencing similar problems and how this had compelled the participant to initiate discussions about the change. Once again, the participant describes how she found the healthcare professional in agreement with the decision.

Jayne, LTHC: ‘with the stuff I found online and when I went and discussed with my diabetologist and he agreed to reduce my metaformin slightly and I have felt better since … It was a forum it was … I found all these people talking about metaformin and their symptoms and I thought yeah that’s happening to me cause you don’t know it’s happening to other people you think it’s normal so by reading it I thought this isn’t alright I’ll go back and tell them.’

In both Emily’s and Jayne’s examples, encounters with internet resources provided the stimulus for decision-making. The resources themselves varied in terms of how they were referred to: ‘girls on Facebook’ versus ‘stuff I found online’ but both were instrumental in prompting our participants to return to their healthcare professionals and to initiate or update their decisions.

For people with STHCs, the stimulating role of internet resources was very apparent. This often focused on a diagnosis decision or an update to a decision around medication.

Taz describes a relatively straightforward health issue and the prominent role of internet resources in decision-making. The information he reads suggests a straightforward diagnosis that is confirmed by the healthcare professional. The very instrumental nature of the resources is described in such a way as to render the ensuing interaction with the doctor almost automated, scripted and perfunctory.

Taz, STHC: ‘Yes, most of the time I’ll probably go in like I’ll say, “I’ve had this and I’ve looked online and I think it’s conjunctivitis.” And most of the time they’ll be like. “Ah yes I think you’re right it’s just conjunctivitis.” So most of the time it’s a case of me knowing what’s wrong or me going to the doctors and like, “This is what’s wrong” and they’re like, “Yes, I’ll give you a tablet.” So yes, online does help for me to know I go in, I say, “I’ve got this” and they’ll agree with me and then they’ll sort me out.’

Jessica describes how information online sparked an instant reaction in terms of a decision around medication. The decision to increase the dose occurs away from contact with any healthcare professional although it is sandwiched between two such interactions. Interestingly, the role of the internet resources is given a low-key feel by Jessica, who cannot recall the specific source of the information at first: ‘I’d gone online’ and even later is unsure, perhaps unconcerned, about the specific name of the website ‘NHS website or something like that’.

Jessica, STHC: ‘Erm, yeah. I had this infection, and I went to a walk-in centre, and they prescribed me the correct medication, but a very, quite low, dosage. And when I’d gone online then, erm, I’d realised that I probably should have been on a double dosage… So I doubled my dose, and then went to my GP, erm, like, on the, the week after, and that was kind of based on the information that I’d got from, like, I think it was like the NHS website or something like that. Erm, so I did that again after I got my antibiotics for the tonsillitis. Erm, just to check.’

In this final example, a decision made in the consulting room between Alex’s Dad and his doctor is updated with immediate effect when Alex (and we presume from the use of the pronoun ‘we’ other members of his family) encounter information ‘online’. The family decide on the basis of that information to alter their father’s medication regime. In this example, the decision-making occurs completely separate from the healthcare professional, they are neither named nor even identified but merely referred to as ‘them’. Reading online information prompted a decision to be made and the healthcare professional is rendered the recipient of the decision outcome rather than being a resource in the process.

Alex, STHC: ‘I mean, my dad got given, erm, his medication… but then we read through all the side effects [online] and we were like, “You should probably not take them.” And we decided to not take them and see if he can manage it normally. Because they were like really severe…. I was like, “You are not taking double.” Like, because he has prepacked medication things, so I went in each one and put it back to half manually. Then told him, I was like, “You’re going to do that now tomorrow. You’re going to whenever you get your appointment, you’re gonna go and tell them you don’t why”.’

### An interactional role: mediates the doctor–patient relationship

In this final category, internet resources play an interactional role, mediating the doctor–patient relationship and, in so doing, impacting upon shared decision-making. In the first example, internet resources play a positive interactional role bolstering the doctor–patient relationship and enhancing communication around decision-making.

Leah describes the way the use of the internet as part of the decision-making process is explicitly acknowledged by her health professional. The doctor uses the fact that she knows her patient will have used the internet (although this is only implied in what is said) ‘you’ve researched that’ to discuss her patients options in some depth, detailing the positives and negatives of different medications.

Leah, LTHC: ‘Yeah because you sort of you know an answer so you’ll deliberately ask it to see if they’ll say the same answer or to elaborate on anything that you’ve just said as well so yeah I definitely do, cause obviously I got that conversation from my doctor about what medications, and I bet you’ve researched that already and yes it’s good for this and no it might not be good for that, erm, and the same when I went for me ultrasound I was able to talk more to the sonographer or… I think that’s what you call them, erm so yeah you feel like because you, you know the answer you still ask it to see if they’re gonna say the same thing as what you’ve, what you’ve read.’

In the remaining two examples, Jake and Mia explain how they feel that internet resources have played a negative interactional role in their relationship with their healthcare professionals. The environment for exchanging knowledge important to shared decision making has been altered by the negative interactional role of the internet.

Jake explains how a previous instance of using the internet hangs over the doctor–patient relationship and has left him in an uncomfortable position. At a previous appointment, the participant had printed off some research on an unorthodox form of pain relief and took it to his doctor to discuss. The doctor made some critical points about the nature of the research the participant had brought along, and this had led to Jake feeling awkward at their subsequent appointments. The quote illustrates how the worsening relationship has led to a situation in which communication around decision making is almost non-existent and shared decision-making is no longer possible.

Jake, LTHC: ‘Yeah so it was just like that he was like well nah it’s probably best we just stick with this cause you’re already on this I was like right okay well and you just kind of agree with them because some of them just don’t involve you much in erm like the decisions and stuff …, I didn’t feel as comfortable with him the next time in all honestly… after we had those papers and stuff erm he… it just felt cold like he’s considerate but erm like whenever that he mentions surgery as an example he’ll say erm like have you given it a thought yet and I’ll be like yeah but I’m not doing it bla bla bla and he kind of like sits there for a split second like I dunno why I bother.’

In the final example, Mia describes an unsatisfactory encounter in which she refers to the doctor’s annoyance about her perceived use of the internet prior to the consultation. The participant describes how the doctor is angry that she has appeared to have already made a decision regarding her own treatment. In the recollection of this event, the participant is keen to deny that she had used the internet to pre-empt her treatment choices. It is unclear as to whether the dismissive reference to ‘googling’ is really a reflection of the doctor’s attitude towards the internet but it does appear to suggest that within the consultation space, the internet has had a negative interactional impact on the doctor–patient relationship and the communication around decision-making (or at least that is how the participant assesses the situation).

Mia, STHC:’I went to the doctor about something more recently, and I was a bit annoyed because she says “Alright, so you’ve already made up your mind”, and she actually said “How do you, how do you want to treat it?” And I hadn’t googled anything to do with treatment, and I was just like “Well, that’s not my job, that’s your job”. I’m just coming in armed with my knowledge that, you know, these are what my symptoms are, because there’s no- I don’t see any reason in going in with, sort of, preconceived ideas about how it’s going to be treated, unless, you know, they say “This is what we do in every case”.’

## Discussion

This study examined the role of internet resources in decision-making, and has shown how three overarching roles play out across a range of both long- and short-term health conditions. Taking a distributed decision-making approach to decision-making activities has allowed us a more nuanced and realistic account of each of the roles as we see them in action over time, in different settings and in relation to different stakeholders. This study has allowed us to go beyond individual health conditions and specific websites to be able to draw broader conclusions about the use of internet resources. We also highlight the important finding that the role of internet resources is not always positive in relation to decision-making.

For some of our participants, internet resources play a supportive role – a decision crutch. The resources offer a form of support for checking, reassuring and confirming decisions already made. For others, internet resources play a stimulating role, a way of using knowledge found online to initiate, sustain or transform a decision. Finally, internet resources play an interactional role, mediating the doctor–patient relationship. This can have both a positive and a negative effect on communication around shared decision-making. That internet resources play a supportive role in decision-making is a finding that resonates with the existing literature. Here, the resources provide a way of allowing people to evaluate and confirm the decisions they have already made rather than initiating decision-making.^28^ This supportive function may occur explicitly, providing ‘reassurance and comfort’ or almost go unrecognized, an unconscious check to validate decisions made. The way in which internet resources can act to sustain decision-making in this way resonates with previous studies suggesting that specific sites including OSGs can provide resources for people evaluating and coming to terms with the decisions they have already made.^1^ Our study extends this finding to internet resources more broadly and indicates that this role is not limited to peer information or specific forums. We see that rather than stimulating decision-making, internet resources act behind the scenes to validate the decision made elsewhere at another time. The decision itself may be as yet unarticulated, or perhaps even unacknowledged by the individual, but once supported in this way comes into sharper focus, and becomes a reality. What is clear from our findings is that this supportive role takes place within a distributed decision-making context so that a decision-making process that started elsewhere is over time further shaped and supported by encounters with internet resources in other settings and often with other stakeholders.

Internet resources also provide people with the stimulus to initiate and transform decision-making. This stimulating role allowed people to recognize that decisions needed to be made. These included new decisions as well as transformations and updates to existing decisions. In these cases, information online prompted people to make a decision or to initiate decision-making. The role of the healthcare professional in this respect varied. In order to sustain decisions, we see patients and healthcare professionals marking agreement with each other’s decisions.^10^ This agreement can be underpinned by internet-based knowledge. As noted in other studies, the healthcare professional is often the validator of information from the internet.^29^ Our participants used internet resources to initiate decisions around medication, treatment, diagnosis and testing. Importantly, they used these resources not only to obtain the knowledge necessary to initiate the decision-making process with the healthcare professional, but also the ‘power’ to do so.^30^ Participants felt confident and emboldened to seek discussion with their healthcare professional, to follow up on a consultation, or to return and seek a certain course of action.

We saw examples of the different ways in which the stimulating role of internet resources worked its way through the decision-making process in relation to discussions with healthcare professionals. In some cases, it formed the basis for discussion towards the decision and, in others, healthcare professionals were simply ‘informed’ of the decision. There were also examples in which decisions were made, and at least initially, sustained without any input at all from the healthcare professional. Here, information derived online stimulated an immediate decision – one that was enacted independently and in which reference to the healthcare professional appeared only to be an afterthought. Again, examining the role of internet resources using a distributed decision-making approach allows us to capture the nuances of the stimulating role, highlighting its function in initiating and updating decisions across time and across settings.

Finally, internet resources perform an interactional role, mediating the doctor–patient relationship and thus impacting on communication around decision-making. Here, reference to the patient using internet resources had an impact on the interaction between patient and doctor and altered the environment for decision-making. In some cases this was a positive change and facilitated a more in-depth discussion about the decision to be made. Often, however, the interactional role was negative, with a reduction in the quality of the doctor–patient relationship leading to an environment that was no longer conductive to good communication around decision-making. The interactional role was sometimes limited to a single encounter and at other times mediated the ongoing relationship.

### Distributed decision-making: focus on internet resources as knowledge sources

Taking a distributed decision-making approach to the role of internet resources is novel and builds upon Rapley’s concept of multiple sources of knowledge.^10^ We see that knowledge, and experiential knowledge in particular, shapes decision-making and emerges through decision-making. Rapley describes using these forms of knowledge to ‘justify, explain, argue against, make sense of, provide evidence for, comment on, agree with, account for – particular decisions’ and indeed we see examples of this in our data with participants drawing upon knowledge and experiential knowledge from internet resources.^10^ Focusing on the internet in this way acknowledges the point made by Clayman et al., who argue that shared decision making often ignores the informational environment to which patients have access.^31^ For many of our participants, identifying how and when they had made a health decision, in fact naming a decision as such, was far from straightforward. The interviews revealed issues or events that emerged slowly or developed over time and, involved discussions with multiple people, and an often non-linear interaction with technology. Participants were often unable to identify specific internet resources, and sometimes webpages, forums and blogs were conflated or simply referred to as ‘online’. In this sense, we note that although certain websites and platforms provide specific types of support, e.g. information, emotional and social support, that in relation to decision-making specifically, the function that the resource performs transcends the importance of the site per se, with different sites providing similar decision-making roles. These struggles with identifying sites and platforms and decision timeframes lend support to the notion of decision-making that is characterized by evolution and transformation rather than a process confined to a single point in within a consulting room.

Overall, we see support for a more closely coupled conceptualization of the role of internet resources in health decision-making in that they are involved in both the initiation of decision-making processes as well as sustaining and transforming decision-making. Internet resources impact upon all aspects of the decision-making timeline, and are important across a range of stakeholder contexts, including the doctor-patient relationship.

### Peer resources specifically

In terms of the types of health resources available online, it is worth reflecting on the role of online peer resources specifically in decision-making processes. As interactive peer-resources in particular become embedded across a wider range of health websites, we note that experiential knowledge featured more heavily in the decision-making narratives of people with chronic and or longer-term health issues. Those with short-term or acute conditions did refer to such sites but more commonly credited information-only sites. Personal experiences provided an affective element, while information-based sites provided basic information, and allowed people to confirm or disprove ‘facts’. Peer-based resources in particular provided a sense of support and reassurance around the decision-making that was particularly important in discussing decisions with healthcare professionals. This supports notions of empowerment, particularly in chronic or serious health conditions in which social and emotional support become more important to individuals.^32^ For some people with long-term conditions, their use of peer resources for support or general information may inadvertently expose them to potential options regarding their health situation. Instead of actively visiting the resources to seek assistance with decision-making, discussions between trusted members of these online health communities might indicate, for example, treatment or medication options, to participants.

For all participants, whether they were making longer-term or short-term health decisions, internet resources were able to play a variety of roles in those decisions: supportive, stimulating and interactional. While both groups used a variety of sites and social media platforms, individuals with more short-term issues reported more use of information sites than peer resources and their integration with healthcare professionals worked in a different way. For these more straightforward decisions, we saw the use of fact-based sites feeding directly into single-event decisions, and fuelling more decisions away from the consulting room. Those with longer-term or ongoing health decisions were more likely to report encountering peer experiences and this was particularly noticeable in the case of pregnancy. Here, participants are typically faced with a standard set of decisions to make across a clear timeline. This means that, at any given point, there are a substantial number of other women in the same position ready to make the same decision at the same time. Personal stories and experiences around pregnancy are often grouped around these timeline points according to due date and are therefore easily accessed and navigated by participants. Overall, however, while the type of sites mentioned or alluded to by our participants varied, the role those resources performed in relation to decision-making was often similar across conditions. As the majority of research on the role of internet resources has focused on longer-term, chronic conditions, these new findings add to the small body of literature on short-term conditions and online resources.

### Integration and shared decision-making

Finally, it is worth considering again the importance of integration within the decision-making process. A distributed approach to decision-making suggests the need for an acknowledgement on the part of both stakeholders (patient and healthcare professional) of the value of different forms of knowledge and its relevance at different times and at different stages of the decision-making process. In this study, whilst we saw many successful forms of knowledge integration we also saw internet resources as a focal point of contention, with the consultation room as a flashpoint for disagreement. The most commonly reported barriers to integrating health information into discussions with the healthcare professional are fear of the doctor’s reaction, embarrassment and concerns over being labelled as difficult.^33–36^ Patients have to have both the knowledge and the power to engage in shared decision-making.^37^ Reading other people’s stories of their own health and wellbeing and empowering individuals to discuss their knowledge, their concerns and priorities through their own and others’ stories should enable doctors and patients to come up with more optimal decision plans.^38^

### Strengths and limitations of the study

Including people with a range of health conditions in the study allowed us to consider how internet resources play a role across a range of decision types not just treatment decisions. This is in line with previous research that has chosen to include a range of different focal health issues to allow a broader consideration of the role of online resources.^1^ Studies examining decision-making have often focused on single health conditions and one key decision around treatment or testing (e.g. France et al.^16^ and Shaw et al.^39^). We wanted to capture a broader range of decisions experienced by patients and get a fuller sense of how internet resources played a role in those decision-making activities. Focusing on a single health condition, however, would have potentially allowed for a longitudinal approach in which participants’ decision-making could be followed over time.^40^ Going forward, an individual condition approach may also highlight specific health decisions or different patterns of internet use in relation to decision-making. Engaging with participants at regular intervals throughout their decision-making activities would allow a closer inspection of distributed decision-making and facilitate the inclusion of additional methods of data collection including observation of patient-doctor communication.^41^ It is important to note that our interviews capture only the participant’s account of any interaction with a healthcare professional. Our ongoing work is examining the professional’s perspective of the decision-making process.

The semi-structured interviews allowed participants to discuss their decision-making in context and to situate their use of internet resources in relation to the different events, times and stakeholders. A future approach might consider other ways of asking people about their use of online resources.^42^ Joining people at the computer while they engage with health information may allow for more insightful greater discussion around motivations and uses. This approach also has the advantage of allowing the researcher to capture a more accurate idea of the different internet resources people use rather than asking people to rely on recall.

The sample consisted predominantly of female participants and, while females are heavy users of the internet in relation to health,^43,44^ recent studies have noted similarities in the way in which men and women respond to health issues such as symptom reporting or consulting with their doctor.^45,46^ Our ongoing work looking at the use of online support groups specifically in relation to decision-making has also seen men and women using the resource in similar ways to support decision-making activities. While gender differences in the types of support exchanged online have been reported, these may be due to the nature of the health condition itself or to methodological differences between the studies.^47^ Finally, this study consisted of an entirely UK-based sample; while healthcare systems vary across countries, engagement with online health resources is high across a number of countries as shown by Tan and Goonawardene’s review.^36^ Interestingly, that review also indicated similarities across countries in the way in which online resources impacted upon the doctor–patient relationship.

## Conclusion

Previous research has indicated that internet resources assist decision-making, but the specific roles they perform across a more distributed decision-making landscape have been understudied. This study has illuminated the ways in which internet resources play a supportive, stimulating and interactional role in decision-making. Internet resources are interwoven into decision-making across time and across encounters with healthcare professionals.
